# Enzymatic recycling of polyethylene terephthalate through the lens of proprietary processes

**DOI:** 10.1111/1751-7915.14114

**Published:** 2022-07-20

**Authors:** José L. García

**Affiliations:** ^1^ Department of Microbial and Plant Biotechnology Centro de Investigaciones Biológicas Margarita Salas, CSIC Madrid Spain

## Abstract

Because society is doing significant efforts to recycle plastics, one option is to break them down into monomers with the help of specialized enzymes. Polyesters such as PLA (polylactic), PCL (polycaprolactone), PHAs (polyhydroxyalkanoates) and PET (polyethylene‐terephthalate) have been considered in more detail for these biological treatments, because they can be now produced as bio‐based polymers, and because ester bounds and esterases are very frequently found in nature. In particular since PET is the most abundant thermoplastic of the polyester family and accounts for approximately 10% of all synthetic plastics on the market, it has attracted more attention. Here we will review the patented biological recycling processes concerning the recycling of PET.

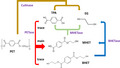

Plastic materials are composed of large polymeric molecules constructed by multiple additions of one type of a small molecule called monomer (homopolymer) or by two or more different types of monomers (heteropolymers). Most plastics in use today are obtained by using monomers derived from fossil fuels and are quite recalcitrant to biodegradation. The term non‐biodegradable applies to plastics that do not break down to a natural, environmentally safe molecule over time by biological processes. The durability of plastics is mainly based on the fact that these molecules are xenobiotics and are, therefore, a rare target for enzymes and microorganisms. Only few marketed plastics are derived from bio‐based monomers and not all of them are biodegradable and/or compostable. In fact, some bio‐based polymers can also be quite recalcitrant to biodegradation or composting as fossil‐derived plastics. The massive use of these plastics recalcitrant to biodegradation and their disposal in the environment is continuously creating serious health and socioeconomic concerns (Ali et al., 2021). Because of that society is making significant efforts to recycle these plastics and give them a new use following the principles of circular economy. In this sense, the reader is directed to a series of articles of the special section of Science “Our plastics dilemma” (Science Vol 373, issue 6550, July 2021).

One option to recycle these plastics, or at least one option to make them edible for microorganisms, is to break them down into monomers with the help of specialized enzymes. In this sense, a large number of studies can be found in the scientific literature concerning the use of enzymes capable of hydrolysing plastics that are not easily degradable, and most of them have been analysed in recent reviews (Amobonye et al., 2021; Danso et al., 2019; Filiciotto & Rothenberg, 2021; Mohanan et al., 2020; Nikolaivits et al., 2021; Prieto, 2016; Purohit et al., 2020; Sales et al., 2021; Satti & Shah, 2020; Wei & Zimmermann, 2017). Through these reviews the reader learns on the scientific state of the art in this field, however, in this article, we pay attention mainly to the knowledge that has been transferred and applied to the industrial sector, by analysing which of these findings has been patented to date. Sometimes, visiting the patent side of science and technology we can find aspects that have not been collected in the scientific journals. Moreover, since patent applications are published a few years before innovations appear on the market, this information can be generally used as a good indicator of things that would soon arrive.

To help us on this matter a recent study has been published by the European Patent Office (EPO) entitled “Patents for tomorrow's plastics: Global innovation trends in recycling, circular design and alternative sources” that registers and analyses the patenting activity worldwide related to plastic recycling and bioplastic technologies (Dossin et al., 2021). Chemical methods have led the field for decades, but EPO report looks at both biological and chemical recycling innovations. Here, we will only focus our analyses on biological recycling processes and particularly in those patented processes concerning the recycling of polyethylene terephthalate (PET) (Table 1).

**TABLE 1:** Non‐exhaustive list of patents related to PET enzymatic degradation

System	Patent	Priority date	Proprietary	Main claim
Cutinase	WO 2000/034450 A1	Dec 4 1998	Novo Nordisk	*H. insolensis* enzyme mutants with higher thermostability produced in *Aspergillus oryzae*.
	CN101792729A	Dec 18 2009	Jiangnan University	*T. fusca* enzyme produced in *E. coli*.
	WO 2012/075662 A1	Dec 8 2010	Jiangnan University	*T. fusca* enzyme produced in *E. coli*.
	WO 2012/099018 A1	Jan 19 2011	Amano Enzyme & Osaka Universtity	*T. fusca* enzyme produced in *E. coli*.
	WO 2014/079844 A1	Nov 20 2012	Carbios	*T. cellulosilytica* enzyme produced in *E. coli*.
	WO 2014/097321 Al	Dec 17 2012	Veeranki, Venkata D. & Hedge, Krishnamoorthy	Cut1 and Cut2 cutinases from *T. fusca* produced in *E. coli*.
	WO 2015/067619 A2	Nov 5 2013	Carbios	Use of enzymes to degrade polyesters.
	JP2015119670A	Dec 24 2013	Kyoto Institute of Technology	Thermostable *Saccharomonospora viridis* enzyme produced in *E. coli*.
	WO 2015/173265 A1	May 16 2014	Carbios	*T. cellulosilytica* enzyme produced in *E. coli*
	WO 2015/097104A1	Dec 19 2014	Carbios	Use of enzymes to degrade polyesters.
	US 2018/0142097 A1	Jun 12 2015	Carbios	Plastic composites with enzymes.
	WO 2017/198786 A1	May 19 2016	Carbios	Amorphization of PET.
	WO 2017/204615 A2	May 26 2016	Universidad Nacional Autónoma de Méexico	*Aspergillus nidulans* enzyme produced in yeasts and *E. coli*.
	WO 2018/011281 A1	Jul 12 2016	Carbios	*Thermobifida* sp. enzyme mutants.
	WO 2018/011284 A1	Jul 12 2016	Carbios	*Thermobifida* sp. enzyme mutants.
	EP 3517608 A1	Jan 30 2018	Carbios	*Thermobifida* sp. enzyme mutants.
	WO 2020/169633 A1	Feb 21 2019	Evoxx Technologies & Henkel AG&Co	*Thermobifida* sp. enzyme mutants.
	WO 2021/181205 A2	Mar 9 2020	Daniel E. Rodriguez	*T. fusca* enzyme mutants with higher activity and thermostability.
	CN112159478A	Sep 28 2020	Jiangnan University	Fusion protein of cutinase and a carbohydrate‐binding module produced in *E. coli* and *B. subtilis*.
PETase	WO 2017/198463 A1	May 17 2016	Henkel AG&Co	Combination of enzyme plus a surfactant.
	CN107794252A	Oct 18 2016	University of Electronic Science and Technology of China	Wild‐type enzyme produced in *E. coli*.
	CN107674866A	Oct 18 2016	University of Electronic Science and Technology of China	Mutant enzyme expressed in *E. coli*.
	WO 2018/168679 A1	Mar 14 2017	Keio University	Combination of mutant enzymes and a surfactant.
	WO 2019/168811 A1	Feb 28 2018	Alliance for Sustainable Energy & University of Portsmouth	Mutant enzyme expressed in *E. coli*.
	CN106367408A	May 11 2018	Tianjin University	Mutant enzyme expressed in *E. coli*.
	CN108588052A	May 11 2018	Tianjin University	Mutant enzyme expressed in *E. coli*.
	CN108467857A	Mar 14 2018	Sichuan University	Mutant enzyme expressed in *E. coli*.
	US 2020/0048621 A1	Aug 8 2018	Kyungpook National University Industry‐Academic Cooperation Foundation	Preparation of crystals of a mutant enzyme.
	CN110241097A	May 24 2019	Shandong University	Mutant enzyme produced in *E. coli*
	CN111057693A	Dec 31 2019	Tianjin Institute of Industrial Biotechnology of CAS	Mutant enzyme produced in *E. coli*.
	CN112852800A	Jan 9 2020	Tianjin Institute of Industrial Biotechnology of CAS	Enzyme with new signal peptide secreted in *E. coli*.
	WO 2021/145822 A1	Jan 16 2020	Agency for Science, Technology & Research	Cyclic PETase with higher thermostability.
	KR102298651B1	May 20 2020	Kyungpook National University Industry‐Academic Cooperation Foundation	Mutant enzyme with higher thermostability.
	WO 2021/148713 A1	Jan 24 2021	VTT Technical Research Centre of Finland	Degradation of PEF.
	CN113774041A	Aug 6 2021	University of Tianjin	IsPETase mutant enzyme.
MHETase	KR102261596B1	Jan 6 2020	Kyungpook National University Industry‐Academic Cooperation Foundation	MHETase mutant with exo‐PETase activity.
PETase & MHETase	WO 2015/025861 A1	Aug 21 2013	Keio University	Enzymes from *I. sakaeinsis*.
	WO 2020/055369 A3	Sep 11 2018	Istanbul Medipol University	Enzymes produced in *E. coli* and *Bacillus subtilis*
	KR102176506B1	Dec 4 2018	Kyungpook National University Industry‐Academic Cooperation Foundation	Enzymes produced in *E. coli*.
	CN113549244A	Jul 16 2021	Dalian Ocean University	Enzymes produced in *E. coli*.
Whole organisms				
*E. coli*	CN106636158A	Oct 20 2016	Tianjin University	Expression of PETase in cell surface.
*Pichia pastoris*	CN106497963A	Oct 20 2016	Tianjin University	Expression of PETase in cell surface.
*P. pastoris*	CN106497964B	Oct 20 2016	Tianjin University	Expression of PETase in cell surface.
*P. putida*	WO 2019/222396 A1	May 15 2018	Alliance for Sustainable Energy & UT‐Batelle	Strain able to metabolize PET.
*E. coli*	CN109402037A	Nov 7 2018	Shenlun Biotech Shenzhen Co ltd	PETase and MHETase are expressed together.
*B. subtilis + (P. putida & Rhodococcus* sp.*)*	CN109929789A	Mar 26 2019	Tianjin University	PETase and MHETase. are expressed together in *B. subtilis* and mixed with *P. putida* and *Rhodococcus* sp. to degrade PET.
*Clostridium thermocellum*	CN111100835A	Jan 7 2020	Qingdao Institute of Bioenergy and Bioprocess Technology of CAS	PETase and MHETase are expressed together.
Mechanoenzymatic	WO 2021/081633 A1	Oct 28 2019	The Royal Institution for the Advancement of Learning/McGill University	Combination of enzymatic and mechanical treatments.

Within the large universe of plastic polymers, polyesters such as PLA (polylactic), PCL (polycaprolactone), PHAs (polyhydroxyalkanoates) and PET have been generally considered in more detail for biological treatments, probably because they can be now produced as bio‐based polymers, and because ester bounds and esterases are very frequently found in nature. In particular, since PET is the most abundant thermoplastic of the polyester family and accounts for approximately 10% of all synthetic plastics on the market (Ali et al., 2021), it has attracted more attention. Although lipases, carboxylesterases and polyester hydrolases can hydrolyse PET, the best hydrolytic yields have been found using cutinases or a mixture of two enzymes, i.e., a PET hydrolase (PETase) (very similar to cutinases) and a mono‐(2‐hydroxyethyl) terephthalate (MHET) hydrolase (MHETase) (Ahmaditabatabaei et al., 2021; Carniel et al., 2021; Carr et al., 2020; Damayanti & Wu, 2021; Maity et al., 2021; Maurya et al., 2020; Salvador et al., 2019; Samak et al., 2020; Tiso et al., 2021; Tournier et al., 2020; Urbanek et al., 2021; Zimmermann, 2020). An additional enzyme (BHETase) will be required to hydrolyse a trace by product bis‐(2‐hydroxyethyl) terephthalate (BHET) (Figure 1).

**FIGURE 1:**
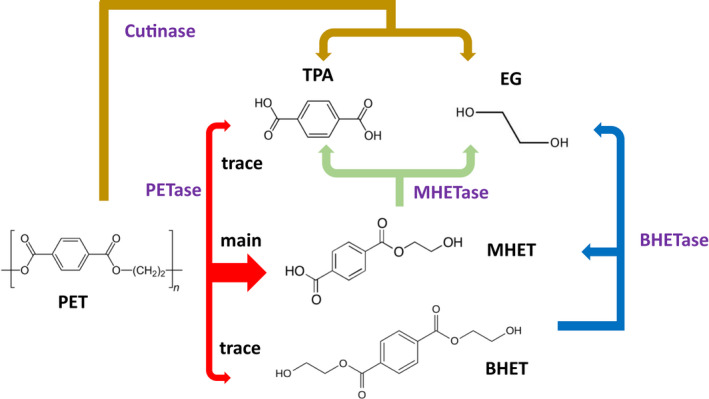
Enzymatic hydrolysis of polyethylene terephthalate (PET). Terephthalic acid (TPA). Ethylene glycol (EG). Bis‐(2‐hydroxyethyl) terephthalate (BHET). Mono‐(2‐hydroxyethyl) terephthalate (MHET)

One of the companies that has been more active in the development of applications of PET degrading enzymes is Carbios (France). In fact, one of the first patents on PET degrading enzymes was filled by this company using a cutinase isolated from the actinomycete *Thermobifida* sp. (Maille, 2015). Carbios also protected other industrial development related to PET degradation using this cutinase with a previous amorphizing of the plastic product prior to depolymerization (Desrousseaux et al., 2017). Carbios also protected the use of a cutinase from *Thermobifida cellulosilytica* expressed in *Escherichia coli* (Boisart & Maille, 2014; Herrero Acero et al., 2015) to break down the crystalline structure of PET and generate high methane potential intermediates (Boisart et al., 2016). However, the treatment of PET with cutinase leads to only 10% hydrolysis after 7 days at 50°C. In addition, in this patent Carbios protected the use of many other hydrolytic enzymes and microorganisms to degrade a large number of plastics (e.g., PLA, polyamides, polyolefines) (Boisart et al., 2016). At the time, it was clear that the hydrolytic yield was not sufficient for industrial recycling, so Carbios worked to develop a new highly efficient recombinant enzyme that, upon publication in Nature, became a breakthrough in the field (Tournier et al., 2020). The new PET hydrolase (e.g., the variant ICCG, F243I/D238C/S283C/Y127G) achieves, over 10 h, a minimum of 90% PET depolymerization into monomers, showing a productivity of 16.7 g of terephthalate/L h (200 g/kg of PET suspension), with an enzyme concentration of 3 mg/g of PET. The new enzyme was developed using a cutinase described by Sulaiman et al. (2012) and the results were translated first to three patents (Topham et al., 2019; Tournier et al., 2018; Zimmermann et al., 2019) opening a new window for its industrial utilization. It is also worth to mention that in a different patent Carbios claims to improve the biodegradability of polyester plastics (e.g., PLA, PCL, PBAT, PHAs and PBS) under environmental conditions by creating plastic composites that integrate one hydrolytic enzyme as well as an anti‐acid filler such as calcium carbonate (Guemard et al., 2018). Interestingly, these findings that protected a new friendlier environmental alternative for plastics have not been published in the scientific literature yet, demonstrating that patents can provide complementary information.

In this sense, it is worth to mention that the first cutinases used to degrade PET were discovered many years ago in *Fusarium solani pisi* (Vertommen et al., 2005) and in *Thermobifida fusca* DSM43793 (former name: *Thermomonospora fusca*) (Müller et al., 2005). Remarkably, the cutinase from *F. solani pisi* had been patented by Plant Genetic Systems (Belgium) for other uses not related to PET degradation (De Geus, 1990). Thermostable variants of a similar enzyme isolated from *Humicola insolensis* were patented by Novo Nordisk and used for PET degradation (Abo et al., 2000). However, although the cutinase from *T. fusca* was discovered in 1999 by Fett et al. (1999), it was not protected at that time. Nevertheless, a recombinant form of cutinase from *T. fusca* produced in *E. coli* was protected by two different patents, one from the University of Osaka and Amano Enzyme Inc. (Japan) (Shigenori et al., 2012) and the other from the University of Jiangnan (China), although curiously the inventors did not claim for any specific use (Chen et al., 2012).

Since these cutinases were not very efficient, new efforts were directed to find new enzymes and thus, Sulaiman et al. (2012) isolated a more efficient PET degrading enzyme from a metagenome library that showed 57%–60% amino acid similarity to *Thermomonospora curvata* lipase and *T. fusca* cutinase. Surprisingly, this metagenomic enzyme was not patented when discovered, however, it was further modified and patented by Carbios, as mentioned above (Topham et al., 2019; Tournier et al., 2018; Zimmermann et al., 2019). Other enzymes that degrade PET and have been cloned and patented are a cutinase from *Aspergillus nidulans* (Farres et al., 2017) and a thermostable cutinase from *Saccharomonospora viridis* (Kawai et al., 2015).

More recently, the interest in PET hydrolases was greatly boosted by the work of Yoshida et al. (2016) who reported that *Ideonella sakaiensis* 201‐F6 was capable of growing using low‐crystallinity PET, thanks to two enzymes that hydrolysed PET and MHET. This finding was patented by the University of Keio (Miyamoto et al., 2015, 2018). Since then, several patents have been filed using these enzymes. For instance, the group of G. Beckham (USA) has developed an intense activity on these enzymes (Austin et al., 2018; Erickson et al., 2021; Knott et al., 2020; Werner et al., 2021). Interestingly, chimeric enzymes fusing the PETase and MHETase proteins with different linkers have been constructed to improve the PET degradation yield (Knott et al., 2020). This group has filled two patents, one shared by the Alliance for Sustainable Energy (USA) and the University of Portsmouth (UK) claiming for a PETase mutant with increasing PET degrading efficiency (Beckham, Jayakody, et al., 2019) and the other shared with UT‐Batelle (USA) claiming for a recombinant *Pseudomonas putida* that expresses PET and MHET enzymes (Beckham, Johnson, et al., 2019). Remarkably, the Agency for Science, Technology and Research (Singapore) has constructed a cyclic enzyme using the SpyTag/SpyCatcher system to increase its thermostability (Ghadessy & Sana, 2021). In addition, other patents have been filled concerning the use of these recombinant enzymes (Demircan & Keskin, 2020; Jin, 2020). Despite the intense work done in recent years with these enzymes, none of these patents have hit the market yet.

## CONCLUSIONS

PET is the most abundant polyester plastic, with almost 70 Mt manufactured annually worldwide for use in textiles and packaging, and thus, the possibility of its continuous recycling is currently considered as a critical challenge. In this review, we have learned that PET biological recycling is getting closer and closer to the market, thanks to improvements in the genetically modified cutinases and PETases/MHETases. In fact, biological PET recycling has been claimed to very close to a happy ending, since a consortium of Carbios, L'Oréal, Nestlé Waters, PepsiCo and Suntory Beverage and Food Europe has announced last July the successful production of the world's first food‐grade PET plastic bottles produced entirely from enzymatically recycled plastic. Bottles have been manufactured based on Carbios' PET enzymatic recycling technology (C‐ZYME® technology) and according to the company the process can be achieved at high speed, i.e., breaking down 97% of PET in just 16 h. Nevertheless, a techno‐economic analysis by Singh et al. (2021) on the enzymatic recycling of PET demonstrates that current processes still consume large amounts of energy and generate some undesirable waste which will require additional efforts to address these and other important limitations. In addition, more research is still required to improve the development of the enzymes.

The enzymatic recycling of PET paves the way for applying this technology not only to other polyesters, but also to all fossil oil derived plastics. If we were able to combine the production of conventional plastics using bio‐based monomers with their enzymatic recycling, we will be closer to envisioning a more sustainable future for plastics. Furthermore, if we were able to substitute these conventional plastics, even if recyclable, by a new generation of plastics that can be easily biodegradable when accidently released to the environment, we will get a cleaner and safer planet.

## CONFLICT OF INTEREST

None declared.
